# Multimodal data-driven multitask learning for enhanced identification and classification of chronic obstructive pulmonary disease: a retrospective study

**DOI:** 10.7189/jogh.16.04028

**Published:** 2026-01-23

**Authors:** Qian Wu, Hui Guo, Ruihan Li, Jinhuan Han, Zhen Zhang, Ayajiang Jingesi, Shuqin Kang

**Affiliations:** Department of Medical Imaging Centre, The Fourth Clinical Medical College of Xinjiang Medical University, Urumqi, China

## Abstract

**Background:**

Chronic obstructive pulmonary disease (COPD), the third leading cause of death worldwide, demands prompt and precise identification and phenotyping for effective management. This study aims to develop a multimodal multi-task learning framework that concurrently performs automated detection and classification of COPD.

**Methods:**

Retrospective multi-task model fusing computed tomography (CT) and clinical data (n = 2320) at a tertiary hospital. Predictive performance for lung-function metrics was assessed using the concordance correlation coefficient (CCC) and mean absolute error (MAE). Classification efficacy was evaluated via the area under the receiver operating characteristic curve (AUC), accuracy (ACC), precision, recall, and F1-score. Generalisability was further verified by replicating the experiments on three distinct backbone networks.

**Results:**

This study included 1624 patients for model training, 348 patients for the validation set, and an additional 348 patients for the independent test set. The optimal model achieved a maximum CCC of 0.75 for forced vital capacity (FVC), corresponding to an MAE of 0.37, and a maximum CCC of 0.77 for forced expiratory volume in one second (FEV1), corresponding to an MAE of 0.33. For the binary classification task (COPD/Non-COPD), the highest AUC achieved through multi-task learning was 0.88, with a maximum ACC of 0.83. In the ternary classification task (COPD/preserved ratio impaired spirometry (PRISm)/Normal), the highest AUC reached 0.87, with a maximum ACC of 0.79.

**Conclusions:**

Multitask-learning models that integrate chest CT images with basic clinical variables outperform their single-task counterparts in both the identification and classification of COPD. This approach supports evidence-based clinical decision-making and advances the delivery of precision medicine.

Chronic respiratory diseases have ranked as the third leading cause of death worldwide since 2010, imposing a substantial burden on global health. Among these disorders, chronic obstructive pulmonary disease (COPD) is the most prevalent and clinically significant. Population ageing is expected to further increase the economic strain on COPD patients. In 2019, annual medical expenditures for COPD in the USA reached 31.3 billion USD and are projected to rise to 60.5 billion USD by 2029 [1]. A large Canadian population-based study reported that COPD patients incurred 48% higher excess health care costs than individuals without COPD [2]. China, bearing the world’s greatest absolute economic burden of COPD, accounts for 83.5% of disease-related economic losses among middle- and high-income countries [3]. Early screening and identification of COPD can therefore prevent disease progression and mitigate both health and economic burdens.

Twenty years ago, researchers observed that some patients presented with respiratory symptoms (*e.g*. dyspnoea) and imaging abnormalities yet did not fulfil traditional spirometric criteria for COPD. A decade later, the Global Initiative for Chronic Obstructive Lung Disease (GOLD) was the first to designate this phenotype ‘preserved ratio impaired spirometry’ (PRISm) and initiated systematic investigation. Across multiple cohorts, PRISm prevalence has ranged from 4.49 to 24.4% [4–6], variations attributable to population characteristics and regional differences. The PRISm phenotype is inherently unstable [7]. Established risk factors for progression to COPD include advanced age, male sex, abnormal body mass index (BMI), and heavy smoking [8,9]. Formal recognition arrived only with the 2023 GOLD, which defined PRISm as a potential pre-COPD state and called for heightened clinical vigilance [10]. An independent risk factor for incident COPD is now recognised to be PRISm [11,12].

However, in real-world clinical practice, approximately one-quarter of patients already diagnosed with, and receiving treatment for COPD do not fulfil the spirometric criteria recommended by GOLD and instead meet definitions of pre-COPD or PRISm [13,14]. Diagnosis of PRISm relies primarily on spirometry; ancillary assessments include chest computed tomography (CT), pulse oximetry [15], and selected biomarkers [16,17]. A post-bronchodilator ratio of forced expiratory volume in one second (FEV1) to forced vital capacity (FVC)≥0.7, with either FEV1 or FVC< 80% of the predicted value, is defined as PRISm.

Pulmonary function test (PFT) remains the gold standard for diagnosing both COPD and PRISm; however, its accuracy depends on high patient cooperation, and under- or misdiagnosis remains common [18]. In primary-care settings, PFT is still underutilised, resulting in delayed or missed diagnoses [19]. Chest CT, which offers the most direct structural information about lung pathology, has emerged as an alternative [20,21]. Yet visual assessment of large CT data sets is inherently subjective and labour-intensive, particularly in individuals with impaired lung function, such as COPD and PRISm patients. Over the past decade, artificial intelligence (AI) has become deeply integrated into health care [22–24], and deep learning – by automatically extracting features from high-dimensional data – has gained prominence in the last two years. These models substantially enhance disease management and reduce clinical workload, achieving notable advances in the diagnosis, staging, prediction, and prognostication of thoracic disease [25–27].

In early-stage CT images, both COPD and PRISm present as small-airway lesions, rendering differentiation difficult when spirometry is unavailable. Yet, the two entities demand markedly different therapeutic strategies. Against the backdrop of mounting global attention to chronic respiratory diseases, non-invasive and readily accessible tools for accurate patient identification and phenotyping are urgently needed. To the best of our knowledge, no multi-task learning framework that integrates chest CT imaging with clinical variables has been developed for the simultaneous identification and classification of COPD. We therefore designed an end-to-end deep-learning model that performs feature extraction and pulmonary-function prediction within a shared-representation architecture. This framework jointly executes binary classification (COPD *vs*. non-COPD) and ternary classification (COPD *vs*. PRISm *vs*. normal). The overarching aim of the present study is to establish a multimodal, multi-task learning pipeline that reliably identifies COPD, thereby providing actionable guidance for clinical management.

## METHODS

Our Institutional Review Board approved this retrospective study and waived the requirement for informed written consent (2025XE0165).

### Participants and data set

We retrospectively enrolled 2720 consecutive patients who underwent both PFT and chest CT at the Fourth Clinical Medical College of Xinjiang Medical University between January 2018–June 2024. Owing to the retrospective design, informed consent was waived. Medical records were reviewed for baseline clinical data and thin-section CT images.

Inclusion criteria:

(1) interval between CT and PFT≤1 month

(2) complete thin-section CT acquisition.

Exclusion criteria:

(1) coexisting thoracic disorders (*e.g*. extensive atelectasis, pneumonia)

(2) suboptimal image quality or artefacts.

Ultimately, 2320 subjects were retained. These were randomly split into a training set (n = 1624), validation set (n = 348), and independent test set (n = 348). All patients were treated as independent subjects and contributed data only once. The complete patient-selection workflow is depicted in Figure S1 in the **Online Supplementary Document**.

Examinations by CT were performed on scanners from Siemens and GE Healthcare. Whole-chest axial images were acquired at full inspiration using 120 kV, 1.25 mm slice thickness; tube current, exposure time, and in-plane pixel size were patient-specific. Spirometric classification followed GOLD guidelines: normal – post-bronchodilator FEV1/FVC≥0.7 and FEV1 (or FVC)≥80% predicted; PRISm – FEV1/FVC≥0.7 and FEV1 (or FVC)<80% predicted; COPD – FEV1/FVC<0.7.

### Data preprocessing

Because CT images were acquired with scanners from different vendors and with heterogeneous acquisition parameters, all volumes were first intensity-normalised. A publicly available U-Net (R231) model, previously validated on several large data sets, was then used to automatically segment the lungs and generate binary masks.

**Figure 1:**
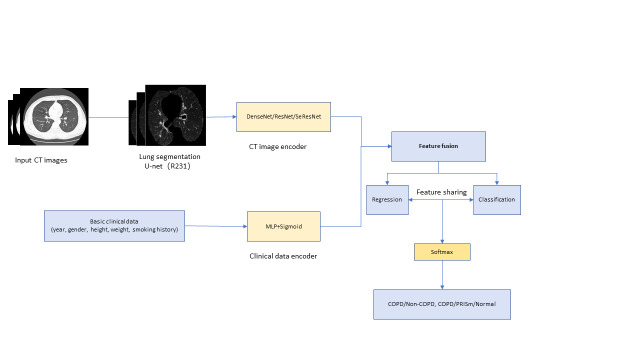
Workflow of the multitask learning model.

To verify the accuracy of the automated segmentation, two radiologists – Guo H (10 years of thoracic CT experience) and Kang SQ (five years) –independently segmented the lungs on 50 randomly selected axial CT scans using 3D Slicer, version 4.10 (Cambridge, Massachusetts, USA), blinded to all clinical data. The resulting masks were reviewed and confirmed by two additional thoracic imaging researchers (Wu Q and Li RH). For anonymisation, Zhang Z and Jingesi A assigned each CT volume a unique, randomly generated identifier and did not participate in image interpretation or segmentation. Inter-observer agreement was excellent, with Dice coefficients of 0.96 (Guo H) and 0.94 (Kang SQ) and an intraclass correlation coefficient of 0.96 between the two readers. The complete workflow of the multi-task learning framework is illustrated in **Figure 1**.

### Model development

The image feature extraction module ingests 3D chest CT volumes and encodes them via a 3D convolutional neural network (CNN) backbone. **Table 1** summarises deep-learning studies dedicated to COPD diagnosis. The majority of models employed CNNs, with densely connected convolutional network (DenseNet) and residual network (ResNet) as the prevailing architectures. Consequently, we selected DenseNet, ResNet, and squeeze-and-excitation residual network (Se-ResNet) as the three backbone models for comparative validation. After successive convolutional blocks followed by global average pooling, the network outputs a 1-D embedding that captures morphological hallmarks such as emphysematous destruction and airway-wall thickening. Basic clinical variables – age, smoking history, and body BMI – are read from a CSV file and projected to the same latent dimension through a Multi-Layer Perceptron. A sigmoid-gated attention mechanism adaptively re-weights these variables, amplifying features most informative for disease discrimination. The re-weighted clinical embedding is then concatenated with the image embedding to achieve cross-modal fusion. The fused representation is further refined by two fully-connected layers, each followed by batch normalisation, rectified linear unit activation, and dropout to enhance expressiveness and curb overfitting. A regression head predicts FEV1 and FVC, while a classification head concatenates the fused features with the regression outputs and feeds them through two additional fully-connected layers to yield disease labels. Detailed model-selection procedures are provided in the supplementary materials.

**Table 1:** Studies on deep learning-based correlation diagnosis for COPD

Author	Country	Model type	Sample size	AUC	Sensitivity	Specificity	Other performance indicators
Chen et al. 2022 [28]	China	CNN (ResNet10) + MIL	4823 (Developmental set: 4552 (NLST: 3715), External test set: 271)	1.00/0.86	0.97/0.0.66	1.00/0.83	ACC: 1.00/0.78
Dorosti et al. 2023 [29]	Germany	CNN (DenseNet121)	78 (Developmental set: 60, Test set: 18)	0.86	NA	NA	
Du et al. 2020 [30]	China	CNN (AlexNet)	280 (10-fold cross-validation)	0.92	NA	NA	ACC: 0.89, TP: 63, FP: 5, FN: 27, TN: 185
Erdem et al. 2023 [31]	Türkiye	CNN (AlexNet)	802	1.00	NA	NA	F1-score: 1.00 Precision: 1.00 Recall: 1.00
Gonza´ lez et al. 2018 [32]	Spain	CNN	9983 (Developmental set: 8983, Test set:1000) (COPD Gene)	0.86	NA	NA	TP: 348, FP: 116, FN: 111, TN: 425
Guan et al. 2024 [33]	China	CNN (ResNet50)	1024 (Developmental set: 726, Test set: 298)	0.90/0.70	0.85/0.63	0.85/0.73	ACC: 0.85/0.64. NPV: 0.53/0.23, PPV: 0.96/0.94,
Ho et al. 2021 [34]	Korea	CNN	596 (5-fold cross-validation)	0.94	0.88	0.94	ACC: 0.89, Precision: 0.83, F1-Score: 0.85
Li et al. 2022 [35]	China	GCN	600 (Developmental set: 500, Test set: 100) (DLCST)	0.81	NA	NA	ACC: 0.77, F1-Score: 0.78, Precision: 0.80, TP: 40, FP: 10, FN: 13, TN: 37
Savadjiev et al. 2021 [36]	Canada	CNN (ResNet50)	274	0.89	NA	NA	Precision: 0.66, Recall: 0.85
Sun et al. 2022 [37]	China	CNN (ResNet18) + MIL	2013 (Developmental set: 1393, External test set: 620 (NLST))	0.93/0.87	0.81/0.80	0.93/0.84	NPV: 0.89/0.98, PPV: 0.87/0.33, F1-Score: 0.89/0.46
Tang et al. 2020 [38]	Canada	CNN (ResNet152)	4742 (Developmental set: 2589 (PanCan), External test set: 2153 (ECLIPSE)	0.89	NA	NA	NPV: 0.76, PPV: 0.85, F1-Score: 0.76, Precision: 0.80
Wu et al. 2023* [39]	China	CNN (ResNet26)	581 (Developmental set: 380, External test set: 201)	NA	0.93/0.92	0.97/0.80	ACC: 0.95/0.85, NPV: 0.93/0.93, PPV: 0.97/0.76
Wu et al. 2023* [40]	China	CNN (VGG-16) + MIL	561 (Developmental set: 360, External test set: 201)	NA	0.95/0.77	0.97/0.89	ACC: 0.96/0.83, NPV: 0.95/0.80, PPV: 0.97/0.88
Xu et al. 2020 [41]	China	CNN (AlexNet) + PCA + MIL	280 (10-fold cross-validation)	0.99	0.99	0.99	ACC: 0.99, F1-Score: 0.99
Xue et al. 2023 [42]	China	CNN (ResNet50) + MIL	1060 (Developmental set: 800, External test set: 260)	0.95/0.87	0.92/0.77	0.92/0.83	ACC: 0.95/0.87
Zhang et al. 2022 [43]	China	CNN (DenseNet201)	599 (Developmental set: 373, External test set: 226)	0.99/0.90	0.95/0.81	0.91/0.84	ACC: 0.93/0.82, F1-Score: 0.95/0.85

ACC – accuracy, AUC – the area under the receiver operating characteristic curve, CNN – nonvolutional neural network, COPD – chronic obstructive pulmonary disease, DenseNet – Densely connected convolutional network, DLCST – Danish lung cancer screening trial, FN – false negative, FP – false positive, GCN – graph convolutional neural networks, MIL – multiple-instance learning, NA – not available, NLST – National lung screening trial, NPV – negative predictive value, PCA – principal components analysis, PPV – positive predictive value, ResNet – Residual network, TN – true negative, TP – true positive, VGG – visual geometry group

*Different studies by the same first author.

The model is trained end-to-end, leveraging the synergy between imaging and clinical data: regression targets serve as auxiliary tasks that regularise the shared representation and improve classification accuracy. In conclusion, the framework simultaneously yields pulmonary-function estimates and disease classification, enabling early screening.

### Statistical analysis

Statistical analyses were performed with IBM SPSS Statistics, version 27.0 (Armonk, New York, USA). Continuous variables are expressed as mean ± standard deviation, and categorical variables as counts and percentages. Continuous variables were compared by one-way analysis of varianc (ANOVA) followed by tukey’s honestly significant difference (HSD) post-hoc test for pairwise correction. Categorical variables were assessed with the χ^2^ test and bonferroni-adjusted for multiple comparisons. Statistical significance was defined at *P* < 0.05.

## RESULTS

### Patient characteristics

A total of 2320 participants were enrolled (1402 men and 918 women). To mitigate class imbalance, participants were randomly allocated to training, validation, and independent test sets via stratified random sampling. **Table 2** summarises their demographic and pulmonary-function characteristics across the training, validation, and independent test sets.

**Table 2:** Patient characteristics

Variable	Training set (n = 1624)	Validation set (n = 348)	Independent test set (n = 348)	*P*-value
Man/female, n (%)	981/643 (60.41/39.59)	210/138 (60.34/39.66)	211/137 (60.63/39.37)	0.10
Age, year	66.51 ± 10.44	66.28 ± 10.68	66.78 ± 10.26	0.81
Height, cm	164.11 ± 8.3	164.2 ± 8.39	164.26 ± 8.25	0.95
Weight, kg	68.36 ± 11.09	68.84 ± 11.19	68.31 ± 11.54	0.75
Never/former/current smokers, n (%)	978/327/319 (60.22/20.14/19.64)	210/69/69 (60.34/19.83/19.83)	210/70/68/ (60.34/20.11/19.54)	1.00
COPD/PRISm/normal, n (%)	1068/298/258 (65.76/18.35/15.89)	231/66/51 (66.38/18.96/14.66)	240/56/52 (68.97/16.09/14.94)	0.77
FEV1	1.71 ± 0.71	1.70 ± 0.71	1.63 ± 0.67	0.16
FVC	2.65 ± 0.79	2.64 ± 0.8	2.60 ± 0.75	0.48

COPD – chronic obstructive pulmonary disease, FEV1 – forced expiratory volume in one second, FVC – forced vital capacity, PRISm – preserved ratio impaired spirometry

Among the COPD, PRISm, and Normal groups, we observed statistically significant differences (*P* < 0.001) in age, sex, height, weight, and smoking history. The COPD and PRISm groups were predominantly male (64.72, and 83.81%, respectively), whereas the Normal group had a notably lower male proportion (15.79%). The PRISm cohort displayed the highest mean height and weight. Never-smokers constituted 88.4% of the Normal group, compared with 56.4, and 50.2% in the COPD and PRISm groups, respectively. Detailed clinical characteristics are provided in Table S1 in the **Online Supplementary Document**. Upon post-hoc testing, all indices differed significantly across groups except for FEV1 between the PRISm and Normal cohorts (Table S2 in the **Online Supplementary Document**).

### Multitask learning classification performance

**Figure 2:**
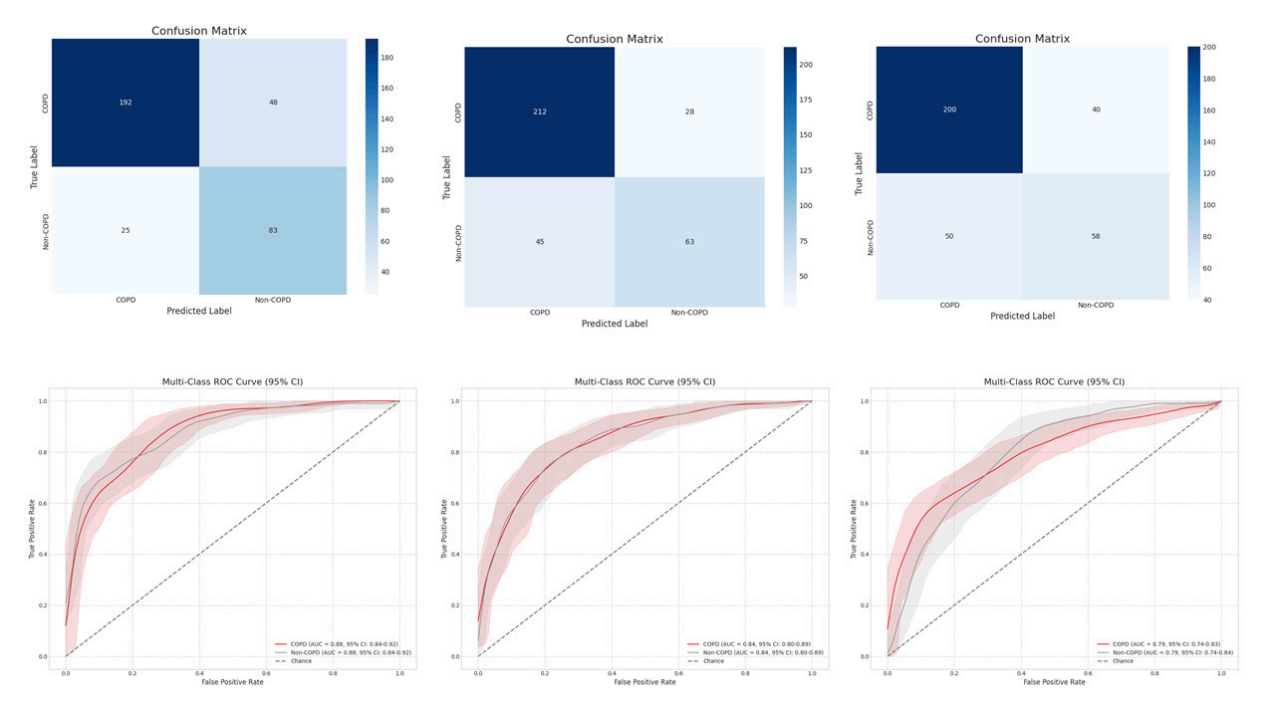
Two-class confusion matrix and ROC curve for multi-task learning. ROC – receiver operating characteristic curve.

**Table 3** summarises the classification performance of the multi-task learning models on the independent test set. In the binary task distinguishing COPD from Non-COPD, DenseNet achieved the AUC of 0.88 and ACC of 0.79, ResNet achieved 0.86 and 0.83, and SE-ResNet achieved 0.79 and 0.74. In the ternary task distinguishing COPD, PRISm, and Normal, DenseNet produced AUCs of 0.87, 0.85, and 0.91 with an ACCs of 0.79; ResNet yielded 0.85, 0.83, and 0.91 with 0.78; and SE-ResNet yielded 0.78, 0.78, and 0.83 with 0.75. Across all backbones, the multimodal binary classification attained a peak AUC of 0.88 and a peak ACC of 0.83, both delivered by DenseNet, confirming DenseNet as the best-performing architecture overall. **Figure 2** displays the confusion matrix and receiver operating characteristic curve for the binary classification task within the multi-task learning framework; the corresponding plots for the three-class task are provided in Figure S2 in the **Online Supplementary Document**.

**Table 3:** Classification performance of multi-task learning models

	AUC	ACC	Precision	Recall	F1-score
Two-category classification (COPD/Non-COPD)					
DenseNet	0.88	0.79	0.88/0.63	0.80/0.77	0.84/0.69
ResNet	0.86	0.83	0.85/0.77	0.92/0.63	0.88/0.69
SeResNet	0.79	0.74	0.80/0.59	0.83/0.54	0.82/0.56
Three-category classification (COPD/PRISm/Normal)					
DenseNet	0.87/0.85/0.91	0.79	0.85/0.58/0.68	0.90/0.50/0.62	0.87/0.54/0.65
ResNet	0.85/0.83/0.91	0.78	0.85/0.58/0.63	0.88/0.50/0.63	0.86/0.54/0.63
SeResNet	0.78/0.78/0.83	0.75	0.80/0.52/0.66	0.88/0.43/0.48	0.84/0.47/0.56

ACC – accuracy, AUC – the area under the receiver operating characteristic curve, COPD – chronic obstructive pulmonary disease, PRISm – preserved ratio impaired spirometry, DenseNet – Densely connected convolutional network, ResNet – Residual network, SeResNet –Squeeze-and-excitation residual network

The calibration curve quantifies the agreement between the model’s predicted probabilities and the observed frequencies. Perfect calibration is indicated by coincidence with the 45° diagonal: curves lying above the diagonal suggest systematic underestimation of risk, whereas curves below denote overestimation. Figure S3 in the **Online Supplementary Document** displays the calibration curves of the DenseNet backbone for the binary task, where the predicted probabilities align equally well with observed outcomes in both COPD and Non-COPD classes. In the three-class task, which suffers from class imbalance, COPD again exhibits the closest correspondence between model-predicted probabilities and actual frequencies (Figure S4 in the **Online Supplementary Document**). On the precision-recall curve, the model attains its highest discriminative capacity for COPD (average precision = 0.92), whereas discrimination of Normal and PRISm is markedly weaker (Figure S5 in the **Online Supplementary Document**). Decision-curve analysis further indicates that the clinical net benefit for COPD substantially outweighs that for the remaining two categories (Figure S6 in the **Online Supplementary Document**).

### Melting experiment

#### Classification rules for predicted pulmonary function indices

To isolate the contribution of the multimodal feature extractor, we replaced the multi-task framework with a rule-based classifier that derives FEV1 and FVC predictions and then computes the ratios FEV1/FVC, FEV1%pred, and FVC%pred. Table S3 in the **Online Supplementary Document** summarises the pulmonary-function prediction metrics: across the three backbones, FEV1 achieved the concordance correlation coefficients of 0.77, 0.73, and 0.65 and mean absolute errors of 0.33, 0.34, and 0.38, respectively; FVC achieved concordance correlation coefficients of 0.75, 0.72, and 0.67 and mean absolute errors of 0.37, 0.40, and 0.42. Predictions for FEV1 were marginally more accurate than for FVC. The derived ratios were then classified according to the GOLD criteria. For COPD *vs*. Non-COPD, the three backbones yielded AUCs of 0.85, 0.84, and 0.81 and an ACC of 0.79 each. In the COPD/PRISm/Normal three-way task, AUCs were 0.85/0.89/0.44 for DenseNet, 0.84/0.88/0.47 for ResNet, and 0.81/0.88/0.49 for SE-ResNet, with an ACC of 0.77 for all models (Table S4 in the **Online Supplementary Document**). Thus, when pulmonary-function indicators alone were used, DenseNet again performed best, attaining an AUC of 0.85 and an ACC of 0.79 for binary classification. In the Bland-Altman plots of spirometric prediction (Figure S7 in the **Online Supplementary Document**), the 95% limits of agreement for DenseNet-derived FEV1 and FVC were 0.84 and 0.92, respectively. The narrower band for FEV1, indicating tighter clustering of predicted values and reduced residual variability, implies superior accuracy.

#### Direct classification based on multimodal data features

In this experiment, we performed direct classification using only the fused CT imaging and clinical features. Table S5 in the **Online Supplementary Document** summarises the results for the three backbone models. For COPD *vs*. Non-COPD, the AUCs were 0.86, 0.84, and 0.75, and the accuracies were 0.78, 0.78, and 0.71 for DenseNet, ResNet, and SE-ResNet, respectively. In the COPD/PRISm *vs*. Normal task, DenseNet yielded AUCs of 0.86, 0.90, and 0.82 with an ACC of 0.79; ResNet yielded 0.84, 0.90, and 0.83 with 0.79; and SE-ResNet yielded 0.81, 0.87, and 0.79 with 0.72. Overall, DenseNet delivered the best direct binary classification performance, achieving an AUC of 0.86 and an ACC of 0.78.

### Feature extraction visualisation

**Figure 3:**
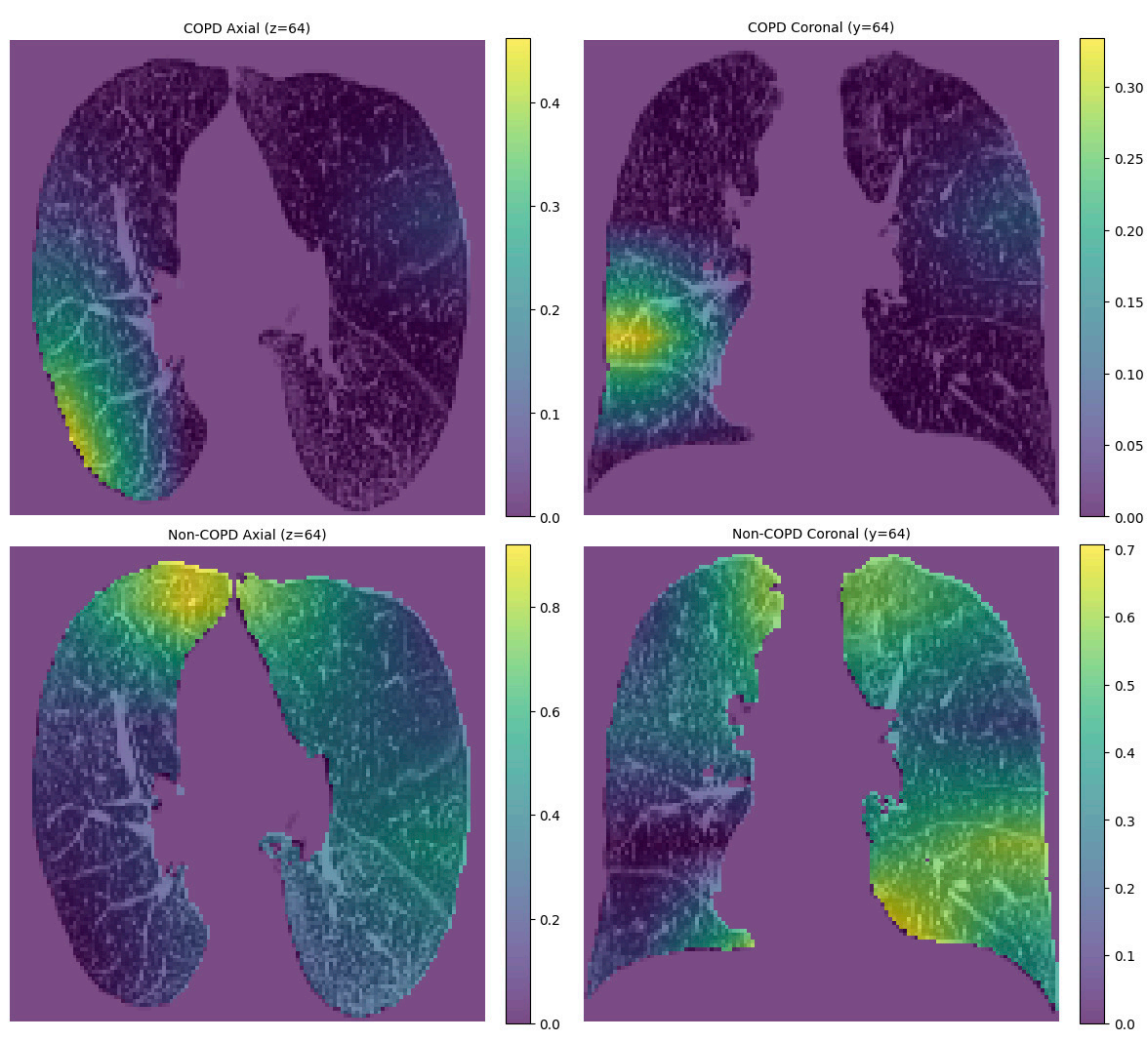
Heat-map visualisations of feature extraction. **Panel A**. The model-predicted probability for COPD. **Panel B**. The probability for Non-COPD. COPD – chronic obstructive pulmonary disease.

Gradient-weighted class activation mapping was applied to generate heat-map visualisations of the extracted features, thereby mitigating the opacity of the AI model. Bright areas correspond to high attention weights that critically influence the prediction, whereas dark areas have little impact. For a 60-year-old male COPD patient, the model attended to emphysema for COPD prediction and to other regions for non-COPD prediction (**Figure 3**).

## DISCUSSION

Accurate identification and phenotyping of COPD are essential for optimal patient management. This study establishes and validates a multimodal, end-to-end multi-task framework that integrates chest CT with basic clinical variables. For patients who cannot perform spirometry, CT scans and accompanying baseline data are fed directly into the model, which then outputs disease labels automatically. The regression head provides FEV1 and FVC estimates, enabling clinicians to rapidly identify high-risk individuals and tailor therapy, while the classification head leverages shared representations to assign COPD, PRISm, or Normal status. DenseNet, ResNet, and SE-ResNet were evaluated to mitigate backbone-specific bias; ablation studies removing either head confirmed that multi-task learning consistently outperforms single-task approaches. DenseNet achieved the best overall performance in both binary and ternary tasks, followed by ResNet, whereas SE-ResNet ranked lowest. Direct classification of fused features marginally surpassed rule-based classification, particularly in the three-class setting, lifting the Normal-group AUC from 0.47 to 0.83.

Given COPD’s high prevalence and low detection rate, CT-based functional inference offers a practical solution. With increasing CT utilisation and successive AI advances, this approach promises improved efficiency and accuracy [44–46]. Our model enables the early identification of high-risk individuals and thus supports timely intervention. Previous studies have shown that advanced age, male sex, abnormal BMI, and heavy smoking are key drivers of progression from PRISm to COPD, while elevated inflammatory markers can also trigger both conditions [47,48]. Consequently, we incorporated age, sex, height, weight, and smoking history as core clinical variables and fused them with pulmonary imaging features for joint disease classification. Evidence further indicates that directly predicting FEV1%pred and FVC%pred yields lower accuracy than deriving these ratios from predicted FEV1 and FVC values, likely because standardised reference equations are poorly adapted to deep-learning outputs or introduce secondary computational errors [49]. Therefore, our ablation experiments predicted only FEV1 and FVC; FEV1/FVC, FEV1%pred, and FVC%pred were subsequently calculated and disease labels assigned according to the GOLD criteria. Previous research has extensively explored diagnostic strategies for COPD [50–52], yet studies employing AI to differentiate COPD from PRISm on CT images remain scarce [53–56]. Existing work is confined to radiomics and conventional machine learning, and no prior deep-learning framework has addressed this distinction. Moreover, limited sample sizes have restricted analyses to binary classification. The present study is the first to introduce a three-class deep-learning model (COPD/PRISm/Normal), aligning more closely with clinical practice. Such AI innovation, coupled with interdisciplinary collaboration, not only refines disease management but also supports junior clinicians, alleviates diagnostic pressure on hospitals, and reduces regional health care disparities [57,58]. Future directions may encompass chest x-ray and magnetic resonance imaging (MRI) modalities [59–62].

In healthy young adults, brisk elastic recoil generates rapid expiratory flow, so the FEV1/FVC ratio normally exceeds 0.70. Applying this fixed threshold systematically overdiagnoses airway obstruction in the young and, conversely, underdiagnoses it in elderly subjects, delaying intervention. Replacing the 0.70 rule with the age, height, sex and ethnicity-specific lower limit of normal (LLN) for FEV1/FVC avoids these pitfalls and aligns diagnosis with individual physiology. Nevertheless, LLN-based interpretation demands complex calculations and is sensitive to the choice of reference equations; consequently, the GOLD report retains the fixed 0.70 threshold as the pragmatic cornerstone of COPD diagnosis. GOLD does, however, strongly recommend that clinicians consult age-specific LLN values – especially in the very young or very old – before finalising interpretative decisions. Future studies will quantify how model performance changes when the fixed 0.70 threshold is replaced by the LLN-based standard and will stratify the analysis by sex, stature and age to identify the subpopulations in which the algorithm maintains its highest accuracy. In addition, given the marked temporal heterogeneity of COPD, we will track prediction stability over multiyear follow-up to determine whether performance drifts enough to require periodic recalibration, an essential step toward reliable lifelong clinical decision support.

Limitations should be acknowledged. First, the retrospective, single-centre design without external validation may limit generalisability; prospective, multicentre cohorts are needed. Second, excluding patients with severe lung disease, incomplete imaging, or suboptimal image quality could introduce selection bias; broader inclusion criteria will be explored. Third, although the model integrates multimodal data, the individual contributions of CT images *vs*. clinical variables have not been dissected; dedicated ablation studies will clarify their relative impact.

## CONCLUSIONS

The multi-task learning framework establishes a fully end-to-end pipeline: raw CT volumes are first auto-segmented, then fused with clinical baseline variables and predicted pulmonary-function metrics within a shared representation, yielding simultaneous disease classification. In summary, our multi-task model surpasses single-task alternatives in COPD identification and phenotyping, delivering clinicians an accurate and efficient decision-support tool.

## Additional material


Online Supplementary Document
