# Supplementary material for: Multimodal data-driven multitask learning for enhanced identification and classification of chronic obstructive pulmonary disease: a retrospective study
Source: J Glob Health. 2026 Jan 23;16:04028. doi: 10.7189/jogh.16.04028 (PMC12828439; doi:10.7189/jogh.16.04028)
Supplement: Online Supplementary Document [file jogh-16-04028-s001.pdf]

Wu Q, Guo H, Li R, Han J, Zhang Z, Jingesi A, Kang S. Multimodal data-driven multitask learning for enhanced identification and classification of chronic obstructive pulmonary disease: a retrospective study. J Glob Health. 2026;16:04028.

Model detail selection

The multi-task network was optimized with Adam at an initial learning rate of 1e-4. Adam unites the momentum and RMSProp estimators, remains robust to sparse gradients and non-stationary objectives, and is comparatively insensitive to the exact learning rate. Input features and regression targets were centred and scaled with StandardScaler. Regularisation was enforced through a synergy of data augmentation, dropout and batch normalisation. Training was halted as soon as validation loss plateaued, and the weights yielding the minimum validation loss were retained.

Figure S1. Dataset establishment diagram

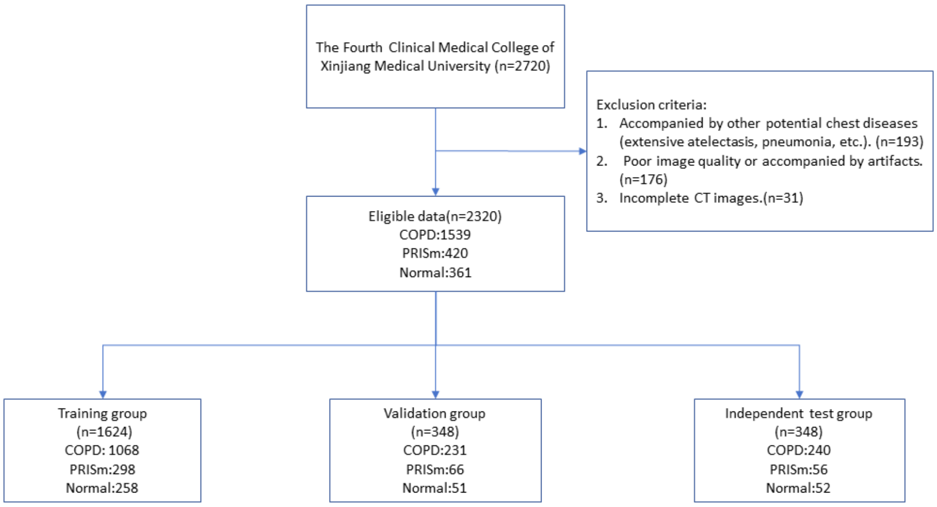

Figure S2. The confusion matrix and receiver operating characteristic curve for the binary classification task within the multi-task learning framework. DenseNet appears on the far left, ResNet in the center, and Se-ResNet on the far right.

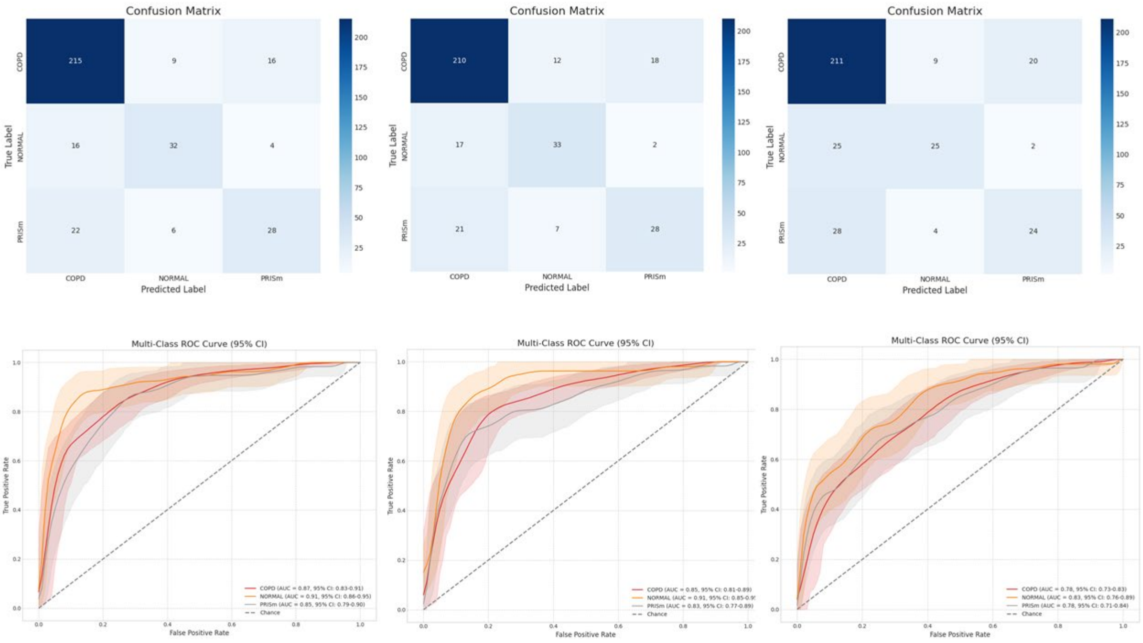

Figure S3. Calibration curves of the DenseNet backbone within the multi-task learning framework for the binary classification task.

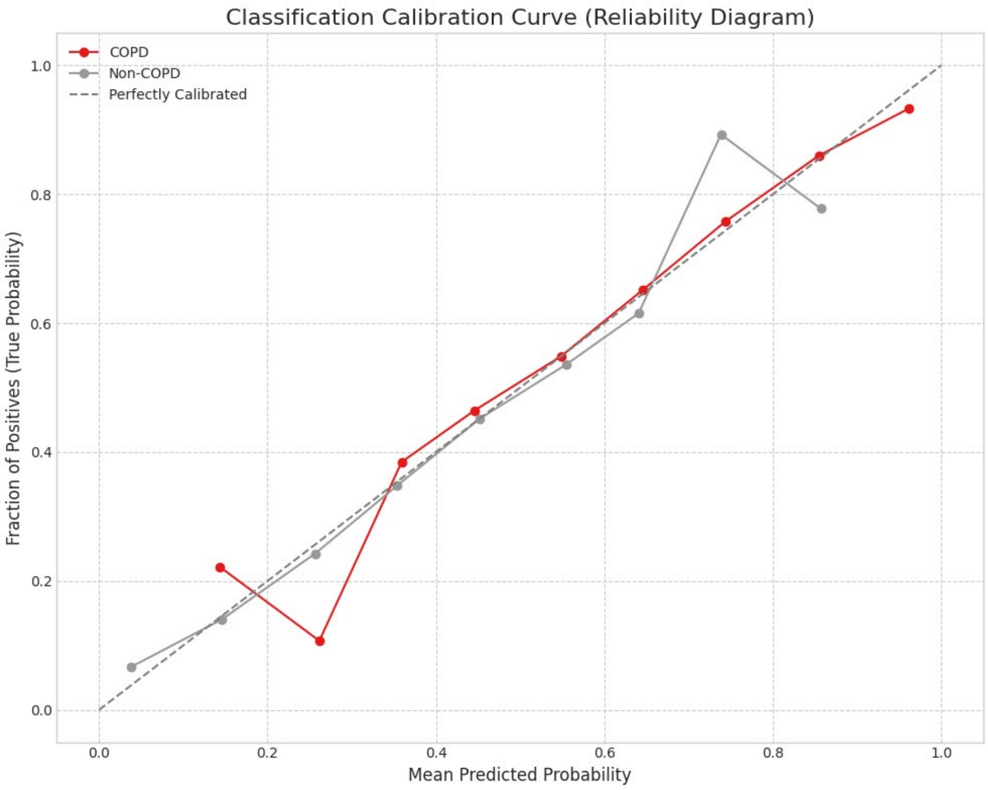

Figure S4. Calibration curves of the DenseNet backbone within the multi-task learning framework for the three-class task.

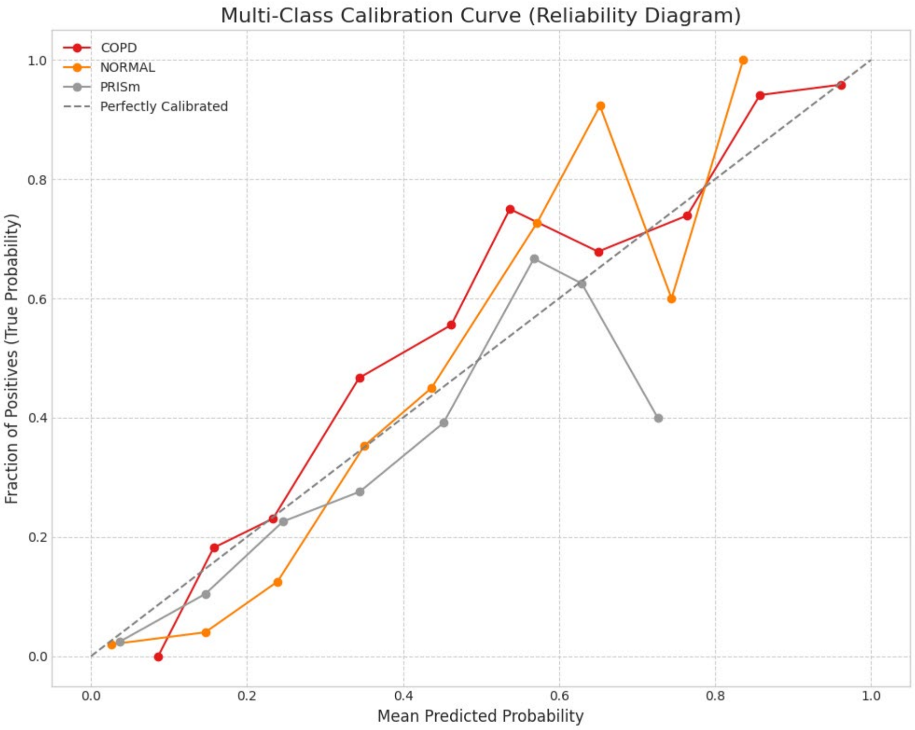

Figure S5. Precision-Recall curves for the three-class task within the DenseNet-backbone multitask-learning framework.

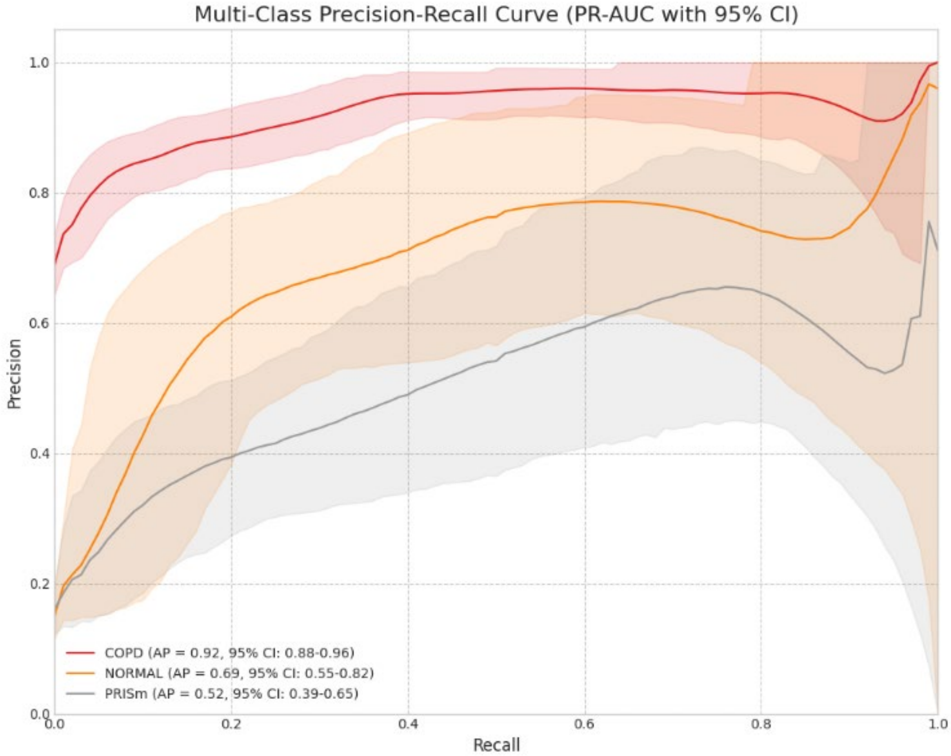

Figure S6. Decision-curve analysis of the DenseNet backbone within the multi-task learning framework.

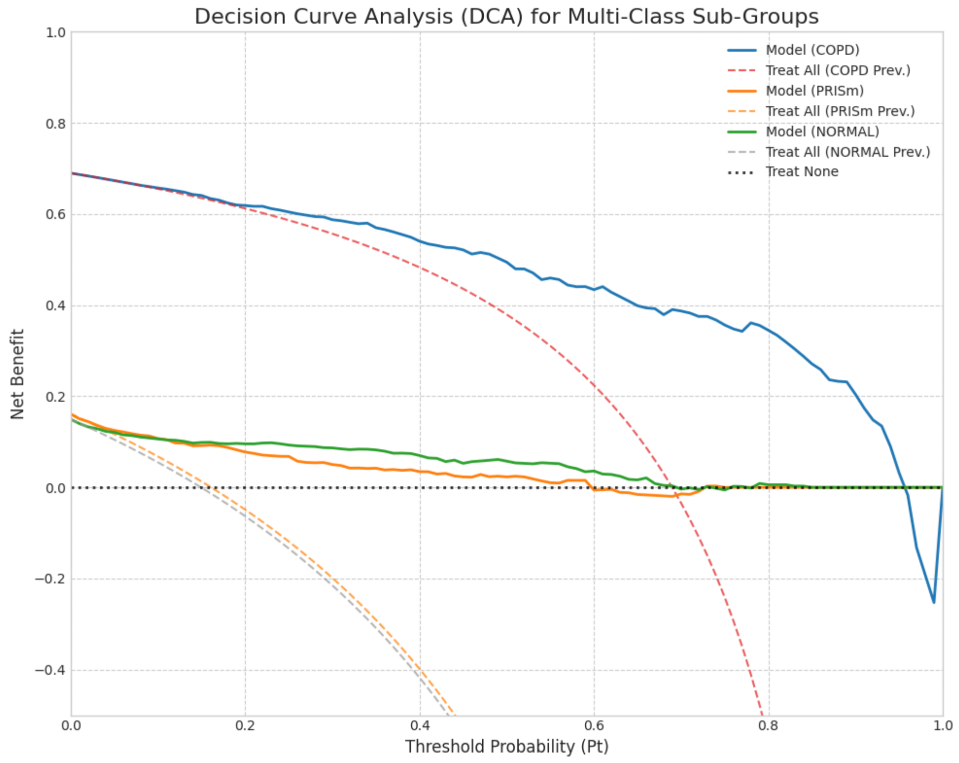

Figure S7. Bland–Altman plots assessing agreement between predicted and measured spirometric indices generated by the regression model.

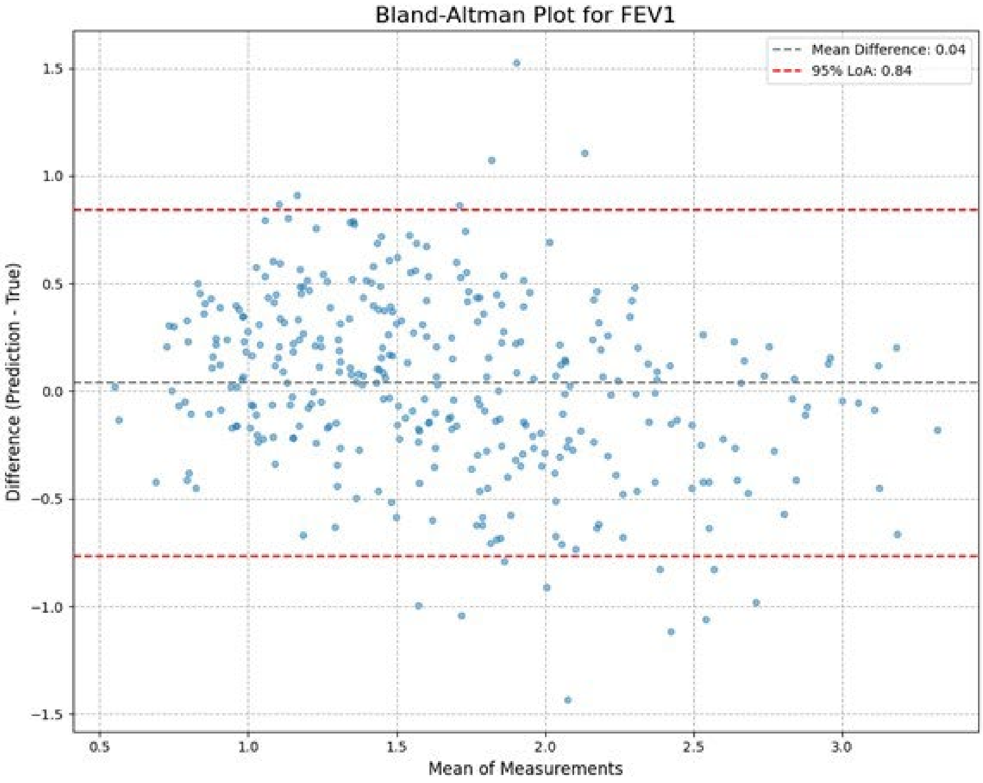

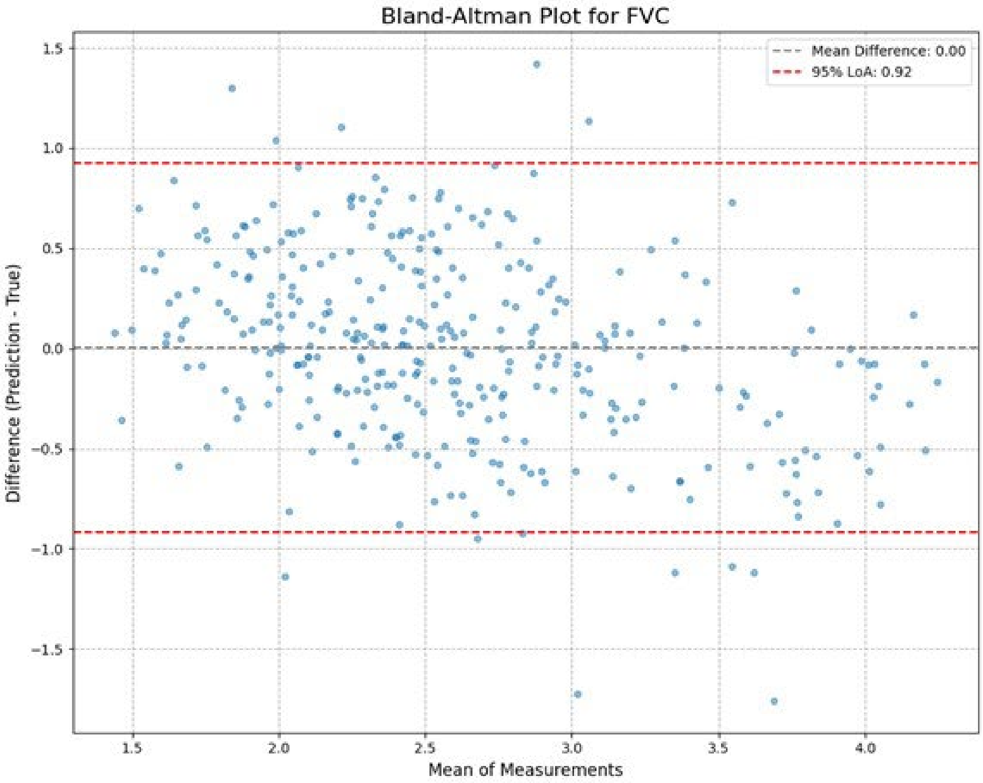

Table S1. Clinical Data for COPD/PRISm/Normal Patients

	**COPD(n=1539)**	**PRISm(n=420)**	**Normal(n=361)**	**P value**

Man/ female, n (%)	996/543(64.72%/35.28%)	352/68(83.81%/16.19%)	57/304(15.79%/84.21%)	<0.001
Age, year	67.91±10.03	65.07±10.95	62.25±10.24	<0.001
Height, cm	164.24±8.45	167.52±7.15	159.81±6.87	<0.001
Weight, kg	68.14±11.24	71.97±11.1	65.51±9.83	<0.001
Never/former/ current smokers, n(%)	868/361/310(56.40%/23.46%/20.14%)	211/89/120((50.24%/21.19%/28.57%)	319/16/26(88.37%/4.43%/7.20%)	<0.001
FEV1, L	1.4±0.55	2.32±0.63	2.24±0.56	<0.001
FVC, L	2.49±0.74	3.02±0.84	2.86±0.69	<0.001

Table S2. Post-hoc multiple-testing correction

**Group**	**P value**						
	**Gender**	**Age**	**Height**	**Weight**	**Smoking status**	**FEV1**	**FVC**
COPD VS PRISm	＜0.001	＜0.001	＜0.001	＜0.001	＜0.001	＜0.001	＜0.001
PRISm VS Normal	＜0.001	＜0.001	＜0.001	＜0.001	＜0.001	0.139	0.006
COPD VS Normal	＜0.001	＜0.001	＜0.001	＜0.001	＜0.001	＜0.001	＜0.001

Table S3. Predictive Performance of Pulmonary Function Indicators

	**DenseNet**		**ResNet**		**SeResNet**	
	CCC	MAE	CCC	MAE	CCC	MAE
FEV1	0.77	0.33	0.73	0.34	0.65	0.38
FVC	0.75	0.37	0.72	0.40	0.67	0.42

Table S4. Rule-Based Classification Performance of Pulmonary Function Indicators

	**AUC**	**ACC**	**Precision**	**Recall**	**F1-score**

Two-category classification (COPD/Non-COPD)					
DenseNet	0.85	0.79	0.67/0.84	0.63/0.86	0.65/0.85
ResNet	0.84	0.79	0.75/0.81	0.51/0.93	0.61/0.86
SeResNet	0.81	0.79	0.73/0.81	0.52/0.91	0.61/0.86
Three-category classification (COPD/PRISm/Normal)					
DenseNet	0.85/0.89/0.44	0.77	0.84/0.64/0.56	0.86/0.65/0.48	0.85/0.65/0.52
ResNet	0.84/0.88/0.47	0.77	0.81/0.61/0.66	0.93/0.52/0.34	0.86/0.56/0.45
SeResNet	0.81/0.88/0.49	0.77	0.81/0.62/0.67	0.91/0.63/0.29	0.86/0.63/0.40

Table S5. Direct Classification Performance Based on Multimodal Data Features

	AUC	ACC	Precision	Recall	F1-score
Three-category classification (COPD/Non-COPD)					
DenseNet	0.86	0.78	0.85/0.64	0.83/0.69	0.84/0.66
ResNet	0.84	0.78	0.82/0.69	0.88/0.58	0.85/0.63
SeResNet	0.75	0.71	0.78/0.53	0.80/0.49	0.79/0.51
Three-category classification (COPD/PRISm/Normal)					
DenseNet	0.86/0.90/0.82	0.79	0.84/0.69/0.58	0.92/0.65/0.39	0.88/0.67/0.47
ResNet	0.84/0.90/0.83	0.79	0.83/0.67/0.68	0.92/0.60/0.45	0.88/0.63/0.54
SeResNet	0.81/0.87/0.79	0.72	0.81/0.56/0.45	0.81/0.67/0.39	0.81/0.61/0.42
